# Contrary trends of grout consumption and water absorption at Sardasht

**DOI:** 10.1038/s41598-025-87884-x

**Published:** 2025-02-07

**Authors:** Mohammad Hassan Nazari, Ali Aalianvari, Mohamed El Tani

**Affiliations:** 1 Mahab-Ghodss Consulting Engineers, Tehran, Iran; 2 Department of Mining, University of Kashan, Kashan, Iran; 3 Rockgro Consulting, Beirut, Lebanon

**Keywords:** Absorption rate, Consumption, Sustainability, Target, Closure, Running limit, Civil engineering, Hydrogeology

## Abstract

A new interpretation of contrary trends of water absorption rate and cement mix consumption in rock grouting is presented. The classical interpretation is either a higher consumption due to fracturing or a higher absorption due to thin fractures that cannot be grouted. Higher grout consumption will depend on the distribution of fractures, their openings and extensions. This is proven by a time simulation of grouting data recorded at Sardasht dam. The simulation is made considering analytic tools and a spreadsheet with computation capabilities. The first step is to prove that the rock has been permeated and the second step is to differentiate the causes of consumption. The minimal flow criterion is integrated to the investigation and is necessary to define a frame for the comparison. Over consumption happens when ignoring the relation between the target and closure.

## Introduction

### Partition

Ewert^1^ emphasizes that preliminary water pressure tests play an important role in implementing grouting in a project. But it is commonly acknowledged, from grouting practice, that there is not a unique relation between water absorption rate and cement mix consumption^2^. Ewert partitioned the outcome of preliminary water pressure tests and cement-based grouting in three groups. The first group includes cases where water absorption rate and cement mix consumption follow the same trend. This group is qualified by Ewert as the group of approximate proportionalities. The second group includes low water absorption rate and high grout consumption. This group is characterized by fracturing during grouting. The third group includes high water absorption rate and low grout consumption. This group is characterized by a hydraulic conductivity connected to numerous thin fractures that cannot be grouted. Indeed, cement suspensions do not enter fractures which openings are smaller than d95. D95 is the particle size below which 95% of the mass of the particle size distribution is found. Penetration of the mix in openings between d95 and 3d95 is possible but limited^3^. A mix is expected to have full penetrability in openings that are larger than 3d95. The limits have been tested experimentally and can vary depending on the conceived experimental apparatus and the mix recipe^4–9^. The upper limit of full penetrability may vary between 2d95 and 4d95. The upper limit increases with flocculation and agglomeration of cement particles. The finer the cement is the larger the flocculation, due to van der Waals and electrostatic forces^10^. The terminology fine, microfine and ultrafine cement refers to a cement which d95 is 40, 20 and 10 microns respectively, according to the European Standards^11^. Repellents and chemical admixtures are added to the mix to impede flocculation, filter cake formation, filtration and bleeding^8,12,13^.

The three groups that have been defined by Ewert are often split in smaller groups and resized. Lopez-Molina et al.^14,15^ partitioned the water absorption rate in three classes, and grout consumption in two classes, resulting in six groups. They emphasized that these groups and their limits are averages of studied cases. This is also the case of Ewert partitions. There are dispersions and deviations from the trends. In Ewert’s Group 1 of approximate proportionalities there is also dispersion. In addition, this group contains cases with contrary trends. This means that there is a separation inside this group with a subgroup of high absorption rate and low consumption, and a subgroup with high consumption and low absorption rate. But the differences in the trend of the subgroups to Ewert’s groups 2 and 3 do not have the same causes. In this note, cases with contrary trends that were observed during the grouting of a cement-based curtain at the Sardasht dam are analyzed. It will be proven that they have permeated the rock. Permeation is a nondestructive grouting of the rock and its fractures. This implies that the injected mix entered the fractures and did not provoke fracturing excluding these cases from Ewert’s groups 2 and 3.

**Fig.1:**
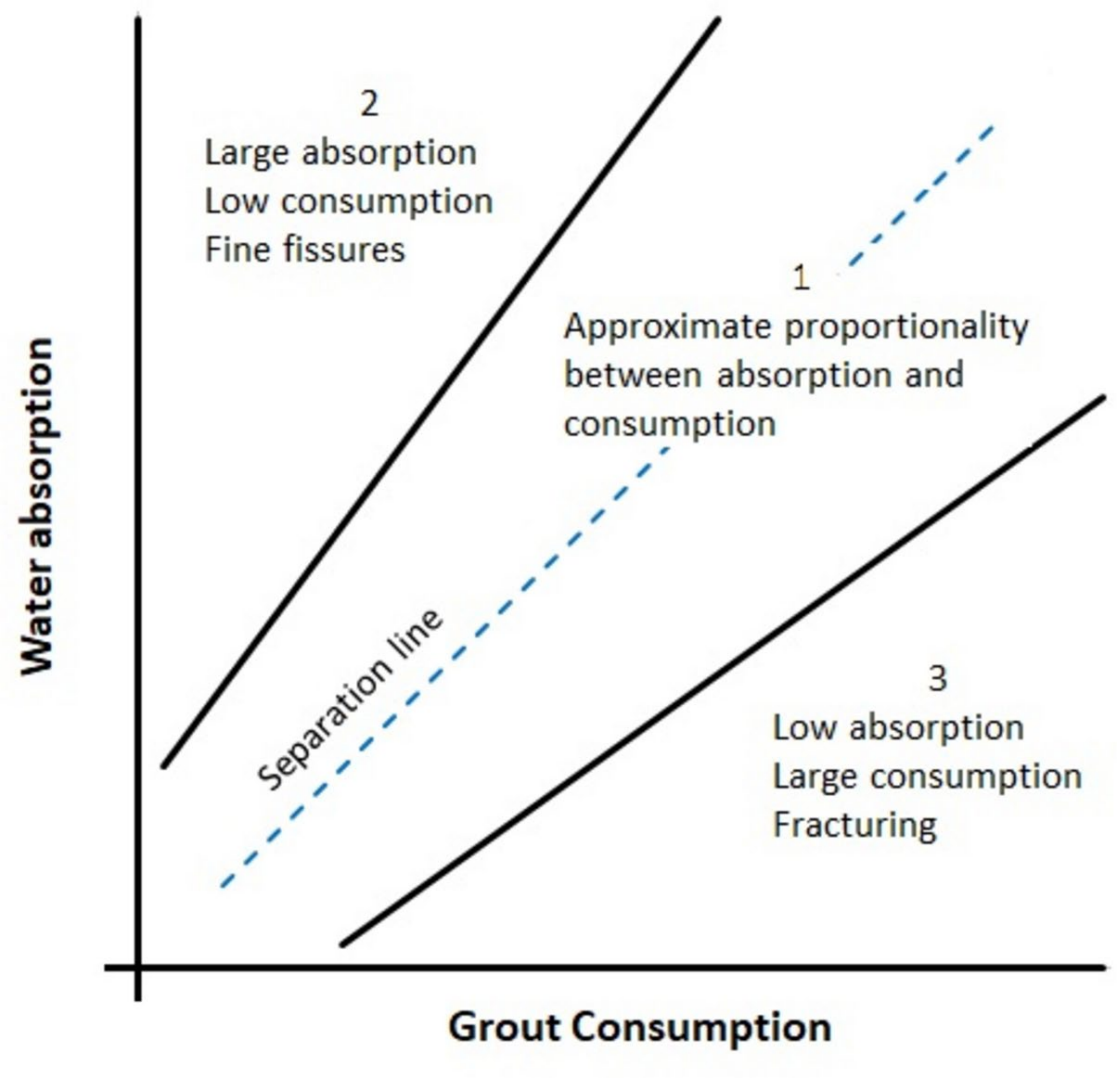
Ewert partition and the separation line that divides group 1 into 2 subgroups.

Figure 1 shows the partition of Ewert in three groups and the separation line that partitions Group 1 into two subgroups. Ewert did not extend the lines to the axes and considers that the domain near the origin of very low absorption rate and consumption is not worth grouting. This domain is characterized by a conductivity that is less than a few Lugeon. A lugeon is a unit of field conductivity that results from the absorption of one liter of water per minute and per unit of stage length, which is injected under a pressure of 10 bars. It has the same unit as the hydraulic conductivity and one Lugeon is 1.667E-7 m/s. There are many conversion values in the literature, ranging between 1.E-7 and 1.667 E-7 m/s. This is due to correction factors^16^ that are related to the geometry of the injection hole. Lugeon tests are also known as water pressure tests and Packer tests. The way to conduct water pressure tests and their interpretations are detailed in many publications, guidelines and normatives^17–19^.

### Analytic tools

The analytic approach to the penetration of a Bigham material, such as cement mix, into discontinuities, has progressed in successive stages. The flow rate equation of a Bingham material injected at a constant pressure in a tube was already known to Bingham^20^ in 1922 and in a channel to Prager^21^ in 1961. Prager’s equation was used for the numerical simulation of a planar network of channels injected with cement mix^22,23^. The radial flow rate equation was harder to obtain due to the singularity of the Bingham constitutive relation in complex geometries^24^. Dai and Bird^25^ ignored the singularity and transformed, empirically, the channel equation of Prager into a radial flow differential equation, using analogies. The equation of Dai and Bird was integrated numerically^26^ to obtain plots in non-dimensional variables of the time advance at a constant grouting pressure.

Many publications^27–30^ cover the details of the analytic development of complex flows and the hierarchical order of approximation that is needed to cope with the singularity of Bingham constitutive relation. The closed form relation for the radial flow rate, which was obtained by El Tani^31^ will be used in this note. The radial flow rate equation in its closed form facilitates greatly the time calculation. Combining the radial flow rate equation in its closed form with the definition of the volumetric rate leads to a first order differential equation. The latter can be integrated exactly in a closed form when grouting at a constant pressure, otherwise numerically using a spreadsheet with computation capabilities.

### Comparison frame

The minimal flow criterion states that grouting is stopped when the flow rate of the injected mix attains a lower limit at a pre-defined pressure. The lower limit is known as the flow rate limit according to the European Normative ^11^. Many variants of the flow rate limit are defined in renown grouting models. These variants are related and can be obtained from one another by simple transformations. In the GIN model of Lombardi and Deere^2^ the variant is called “Penetrability” and in the Amenability theory of Naudts^32^ “Apparent Lugeon”. The Amenability theory is a grouting model that is extensively used in North America according to Bruce^33,34^. The lower limit for the flow rate, and consequently for the penetrability and apparent Lugeon, is stated empirically as by USACE^35^ or based on the grouter’s experience. El Tani and Lopez-Molina^36^ transformed the status of the minimal flow criterion from empirical to deterministic. The transformation was obtained by quantifying the flow rate limit, and defining it as the calculated flow rate at the target. This definition transformed the flow rate limit into a function of the grouting pressure, target, opening of the fracture, radius of the grout hole, viscosity of the mix and its yield stress. The target is the distance that the mix must penetrate in the rock to secure an overlapping with the mix that comes from the neighboring grout holes. The overlap will be previously defined by the designer, so that at closure, the grouted structure has the desired characteristics^37,38^.

In this note, the deterministic form of the minimal flow criterion is used. This implies that a target has to be defined and will be shared by grouting stages of neighboring grout holes. The other shared parameters are the mix properties, the radius of the grout hole and the stage length. Identifying and defining clearly the shared parameters is necessary for a reliable comparison of the outcomes of grout consumption and their subsequent sorting.

## Sardasht dam

### Overview

Sardasht dam is a rockfill embankment dam with a height of 113m and a crest length of 275m. The crest elevation is 1041 masl and the reservoir normal storage level is 1027 masl. The freeboard has a length of 14m and was designed considering a 1000-year flood return period and a 100-year wind return period.

**Fig. 2:**
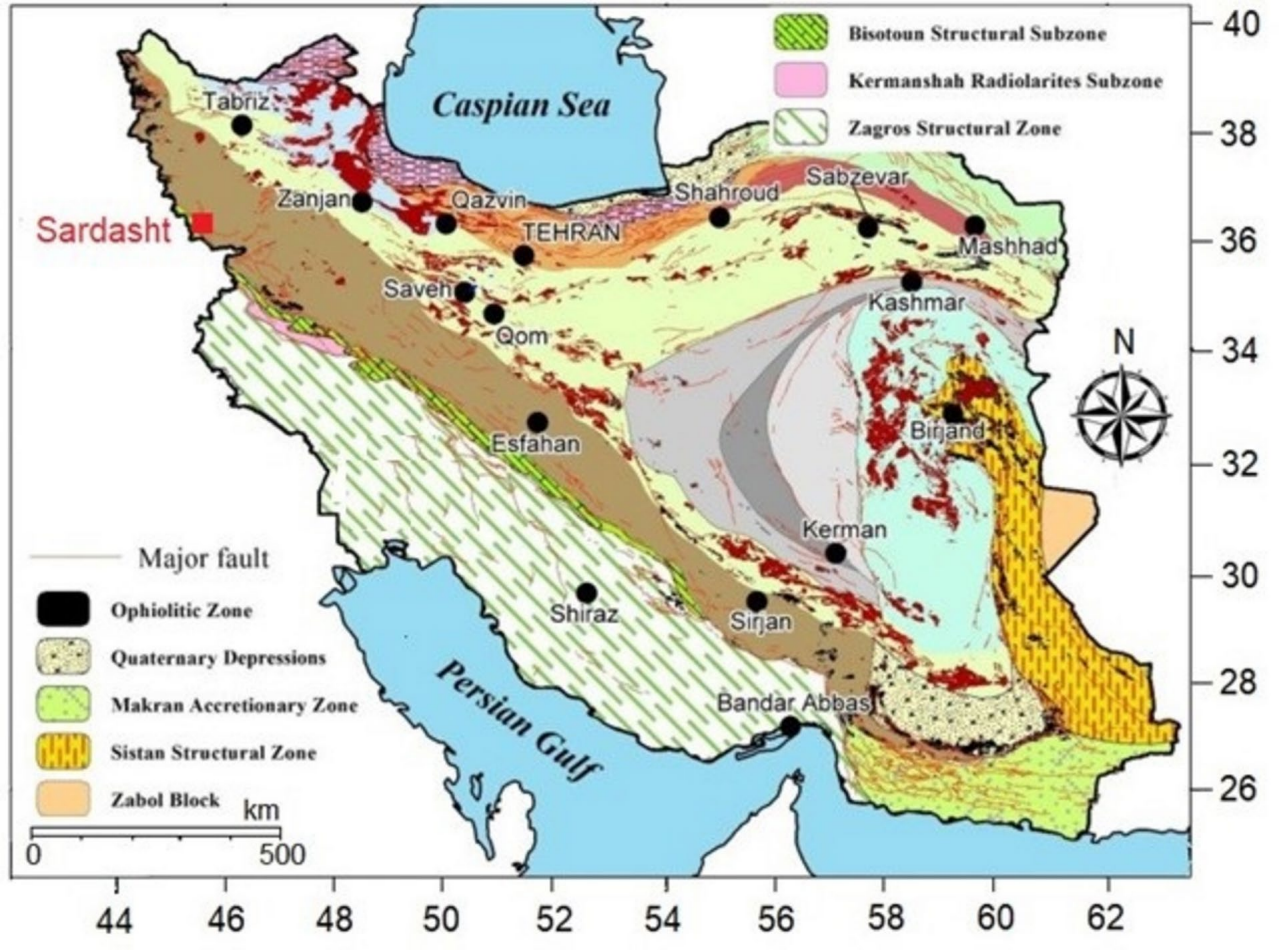
Structural geology map of Iran (adapted from Fazli et al.^39^).

Sardasht Dam was built on the Zab River in the metamorphic belt called Sanandaj-Sirjan. The dam is located in northwest Iran as shown in the structural geology map^39^ in Fig. 2. The foundation of the dam is made of dark Phyllite among an area of a slate rock with siliceous veins and inter-beds of metamorphic sandstone. Slate rock is characterized by foliation of tectonic origin and high frequency narrow joints^40^. Rock masses in the near-surface are weathered and weak. Weathering is reduced below 15m depth and rock mass strength increases.

### Grout curtain

Grouting of a cement-based curtain beneath the core of an embankment dam is necessary to reduce water loss, erosion and piping due to seepage. Remediation, according to Houlsby^37^, is to reduce the hydraulic conductivity beneath the core to between 1 and 3 Lugeon. If none of the mentioned effects due to seepage is a determining factor regarding cost and effect on the long-term stability of the dam, the hydraulic conductivity will be reduced to between 3 and 7 Lugeon. At Sardasht site, the hydraulic conductivity attains more than 23 Lugeon between 30 and 35 m depth^41^. So, grouting is mandatory.

A one-line cement-based curtain was grouted. Cylindrical holes were drilled at equal distance along a line. These are the primary holes and are grouted first. Secondary holes were then drilled and grouted in between at mid distance between the primary holes. Higher order holes, up to the 5^th^ order, were drilled and grouted at mid distance between the existing holes. No hole was grouted at once. Every hole is fractioned in segments, which are successively isolated with packers and then grouted: this is known as “stage grouting” and every stage is determined by its depth. The ratio of water to cement in the mix is 2:1 to which has been added 1% superplasticizer.

### Groutability

Groutability is the ability of a cement suspension to penetrate a fracture. It was mentioned in section "Partition" that a cement mix does not enter a fracture the opening of which is less than d95. Its penetrability is limited when the opening is between d95 and 3d95.

A preliminary water pressure test and a count of the fractures are necessary to determine the stage groutability. In the case where there are many conductive fractures, the two extreme hydraulic scenarios are either a dominant fracture or many fractures of equal opening. This is due to the cubic behavior of the hydraulic conductivity. In the case of a dominant fracture the hydraulic conductivity is

1 $${\text{k}} = \frac{{2\upgamma _{{\text{w}}} {\text{H}}^{3} }}{{3\upmu _{{\text{w}}} {\text{L}}}}$$

in which k is the conductivity, H half of the opening of the fracture, μ_w_ water dynamic viscosity and γ_w_ water specific weight, and L the stage length. Water specific weight is 10 kN/m^3^, water viscosity is 1 mPas and the stage length is 5 m. At Sardasht, Ordinary Portland Cement was used with a d95 of 60μm; d95 = 60μm. A sufficient condition for the groutability of a fracture is that the hydraulic conductivity exceeds 5.8 Lugeon; this quantity is obtained from Eq. (1) considering an equivalent hydraulic opening of 3d95, that is H = 90μm. If this condition is not satisfied, cement consumption is limited, or not possible. In the latter case, forcing will lead to fracturing or jacking. When there are equally conductive fractures, the resultant hydraulic conductivity is obtained from Eq. (1), multiplying the right-hand side by the number of fractures.

### Borehole S2

A borehole matriculated S2 that has exhibited contrary trends of water absorption rate and cement mix consumption is considered in this note for a detailed analysis. Grouting was carried out from the bottom to the surface in stages, most of which were 5 m long.

Before each grouting stage, a water pressure test was performed following a five-step procedure. The injection pressure was increased and then decreased according to the cycle 1, 3, 5, 3, 1 bar, with each step lasting 5 min. Houlsby^42^ classified the behavior of the water flow during the pressure cycle in five classes: Laminar, turbulent, dilation, wash-out and void filling. Table 1 shows the Lugeon conductivity at each stage, and the corresponding Houlsby’s class. The flow is laminar at stages 10–15, 20–25 and 30–35. It is turbulent at stages 15–20 and 20–25. The hydraulic conductivity is less than 2 Lugeon at stages 2–6 and 6–10 that are quasi-impervious. There was no wash-out or void filling.

**Table 1:** Conductivity and consumption at borehole S2; L, T and D refer to Laminar, Turbulent and Dilation.

Stage (m)	2–6	6–10	10–15	15–20	20–25	25–30	30–35
Lugeon test interpretation	L	D	L	T	T	L	L
Conductivity (Lugeon)	< 2	< 2	6	5	4	15.4	23
Consumption (Kg/m)	9.6	7.6	4.2	2.3	4	44	9.2

**Fig. 3:**
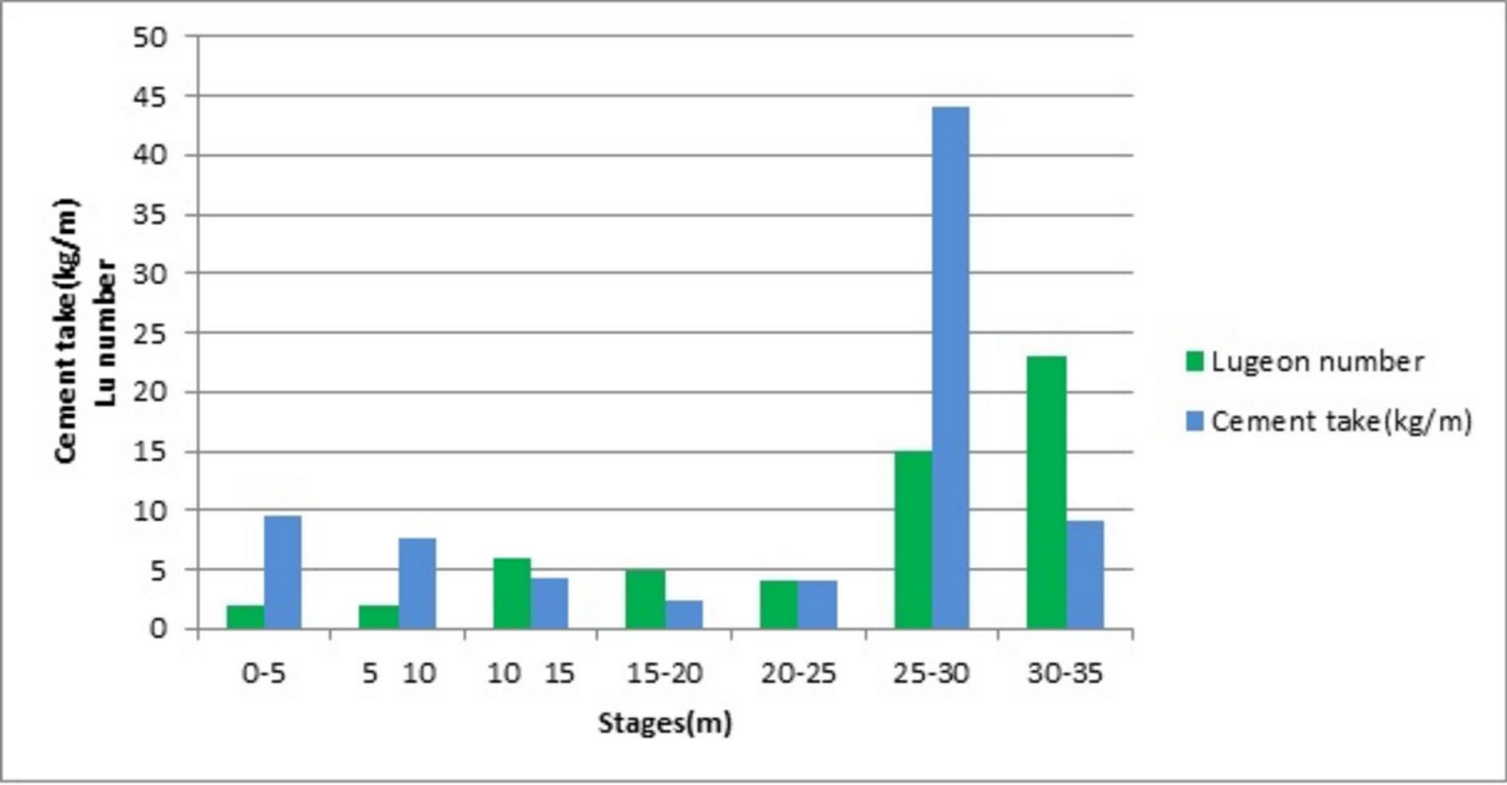
Lugeon and cement take at the different stages of the S2 borehole.

Figure 3 compares the stages’ Lugeon conductivity and grout take in Borehole S2. The hydraulic conductivities of the upper 5 stages are less than 6 Lugeon, which is the threshold for groutability with the cement that has been used. Pressurizing these 5 stages according to Ewert results in fracturing or clogging. The remaining two stages 25-30m and 30-35m satisfy the necessary condition of permeation. Their conductivities are respectively 15.4 and 23 Lugeon. The stage 30-35m has a higher conductivity than stage 25-30m, but its consumption is lower. The consumption of stage 25–30 is 44kg/m and of stage 30–35 is 9.2kg/m. The cement consumption per unit stage length of stage 25-30m is more than four times the consumption of stage 30-35m. These contrary behaviors of absorption and consumption do not necessarily fall within Ewert’s group 2 of fracturing or group 3 of thin fractures that cannot be grouted. A proof is needed that they have permeated the rock and fall within Group 1.

## Time analysis

### Grouting record

The grouting at Sardasht dam has been monitored, keeping records every 10 s of the grouting pressure and flow rate. The recorded data is used in this section for 1-a back analysis of the grouting of stages 25–30 and 30–35, 2-apprehend the contrary trends of their absorption and consumption, and 3- point out excess consumption. Beforehand, an analysis of the preliminary water pressure tests, which considers the counted number of fractures, needs to be performed.

### Conductive fractures

The hydraulic conductivity at the working stage results from the contribution of every conductive fracture that crosses the stage. The extreme scenarios are those where there is one dominant fracture or many fractures that contribute equally. The number of counted fractures of stage 20–25 is five. There are 3 open fractures and 1 tight. The number of counted fractures in stage 30–35 is seven. There are 5 open fractures, 1 tight and 1 filled. Table 2 gives the details of the number of fractures and the measured conductivities.

**Table 2:** Counted fractures.

Stage	Joints			Conductivity
m	Open	Tight	Filled	Lu
25–30	3	1	–	15.4
30–35	5	1	1	23

The counted fractures are not necessarily all conductive. The conductive ones generate the observed hydraulic conductivity. Depending on the number of conductive fractures, the opening of the fractures must be such that the resulting conductivity is the measured one. The openings are obtained, by inverting Eq. (1) and inserting, the ratio of the measured lugeon conductivity to the number of conductive fractures, on the right-hand side. Table 3 shows the openings of the conductive fractures in case there is one or many identical fractures. The opening decreases when the number of conductive fractures increases. The numbering is stopped where the opening is close to 2d95. There are 4 possible configurations in stage 20–25 and 5 in stage 30–35. The subsequent analyses will determine among the configurations which one prevails, that is to find the effective number of conductive fractures at every stage.

**Table 3:** Sets of equal hydraulic openings that generate the measured conductivity.

Stage	Number of fractures	1	2	3	4	5
25–30	Opening (μm)	249	197	157	124	-
30–35	Opening (μm)	284	226	179	142	113

### Governing equations

The main tool of the back analysis is the flow rate equation of the radial penetration of cement mix. The radial flow rate equation ^31^ for a pressure driven flow in a fracture that is injected with a Bingham like material is

2 $${\text{Q}} = \frac{{2\uppi }}{{3\upmu }}\frac{{{\text{H}}^{3} {\text{P}}}}{{\ln \left( {1 + \frac{{\text{d}}}{{\text{a}}}} \right)}}\left( {1 - \frac{{{\text{cd}}}}{{{\text{PH}}}}} \right)^{2} \left( {2 + \frac{{{\text{cd}}}}{{{\text{PH}}}}} \right)$$

in which Q is the flow rate, P the grouting pressure, μ the dynamic viscosity of the cement mix, c the yield stress, a the radius of the grout hole, 2H the fracture opening and d the distance of the advancing front to the grout hole; d is also known as the spread, penetration and advancement. In case there are many equally conductive fractures, the total flow rate is obtained by multiplying Eq. (2) by the number of conductive fractures.

The time advance of the grout is calculated, using the definition of the volumetric rate that is

3 $$\frac{{{\text{dV}}}}{{{\text{dt}}}} = {\text{Q}}$$

V is the volume of the injected mix. For a radial spread, V is the product of the opening of the fracture and the area of a disc, the inner boundary of which is the edge of the grout hole and the outer boundary is the front of the cement mix

4 $${\text{V}} = 2{\text{H}}\uppi \left( {\left( {{\text{d}} + {\text{a}}} \right)^{2} - {\text{a}}^{2} } \right)$$

The unknown variable is the spread d, the initial value of which is d_0_ = 0. The integration of the system of Eqs. (2) to (4) necessitates a boundary condition, which is the grouting pressure P(a,t) at the entry of the fracture. The grouting pressure is also called the “driving action”.

In this note the driving action is the grouting pressure. Combining the system of Eqs. (2) to (4) leads to a first order differential equation that can be integrated exactly and in a closed form when the pressure is constant. For a continuously changing pressure a numerical integration is performed, replacing the time derivative by a finite difference approximation, using the forward Euler method. All of the integration procedure has been programmed using a spreadsheet with computation capabilities. The following schematic procedure was programmed. 

At time t_i_ the flow rate, pressure, spread, fracture’s opening and spread are Q_i_, P_i_, d_i_, H_i_ .

- Calculate Q_i_ using Eq. (2); nQ_i_ is the total flow rate; n is the number of conductive fractures
- Calculate the volume at time t_i+1_ as V_i+1_ = V_i_ + (t_i+1_—t_i_) Q_i_
- Extract the spread d_i+1_ algebraically from Eq. (4)
- Increase i by one unit and go back to the first step

### Back analysis

The scope of the back analysis is to prove that the fractures have been permeated. The proof necessitates to find the number of conductive fractures and their openings and to calculate the flow rate, using the measured grouting pressure as the driving action. The calculated flow rate is then compared to the measured flow rate. It will be recalculated up to the moment it fits the measured flow rate. At every run, the number of conducting fractures, their opening and properties of the mix are changed. The time calculation starts when the grout filled and pressurized the stage hole. The hole’s diameter is 76mm, length 5m and volume 22.7 L. The filling of the hole takes approximately 110 s.

#### Stage 25–30

**Fig. 4:**
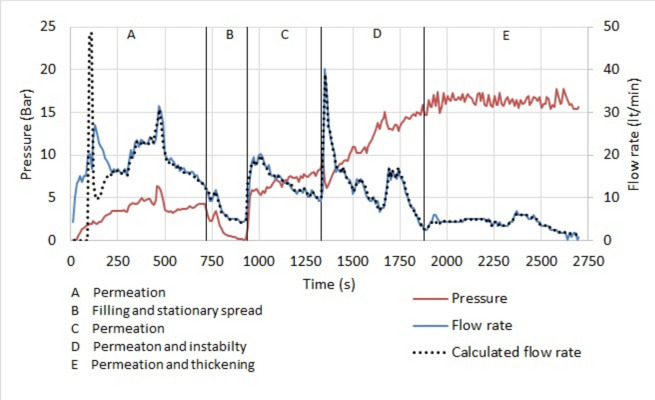
Grouting pressure, measured flow rate and calculated flow rate of stage 25–30.

The grouting pressure of stage 25–30 and the measured and calculated flow rate are shown in Fig. 4. Five periods are indicated in the figure. In periods A, C, D and E the permeation is underway. In Period B there is conceivably a filling of an unexpected cavity near the borehole, which slows down permeation. In Period D, there are instabilities at and near the borehole, like filter cake removal, fracturing or jacking. In period E the mix at the front starts to thicken, increasing the resistance.

The parameters of the back analysis are shown in Table 4. There are 3 conductive fractures with an opening of 155 mm in period A and B, 2 with an opening of 160 mm in period C, and 1 with an opening of 150 mm in period E. The viscosity and the yield stress are also indicated. The viscosity increases steadily from 1.4 to 2.6 mPas, then accelerates in the last period.

**Table 4:** Parameters of the back analysis of stage 25–30.

Period	–	A and B	C and D	E
Time	s	110–960	960–1650	1650–2690
Number	–	3	2	1
Mean Opening	μm	155	160	150
Viscosity	mPas	1.4	2.2	7
Yield stress	Pa	0.2	0.2	0.2

The calculated average opening of the fracture is in good agreement with the analysis of the water pressure tests, summarized in Table 3. It was projected that for 3 conductive fractures their hydraulic openings would be 157 μm each. The deviation of the calculated openings of the back analysis to the hydraulic opening is 4 μm. The deviation is the square root of the sum of the square of the difference of the calculated opening and the hydraulic opening, multiplied by the number of fractures in each period, divided by their sum.

#### Stage 30–35

**Fig. 5:**
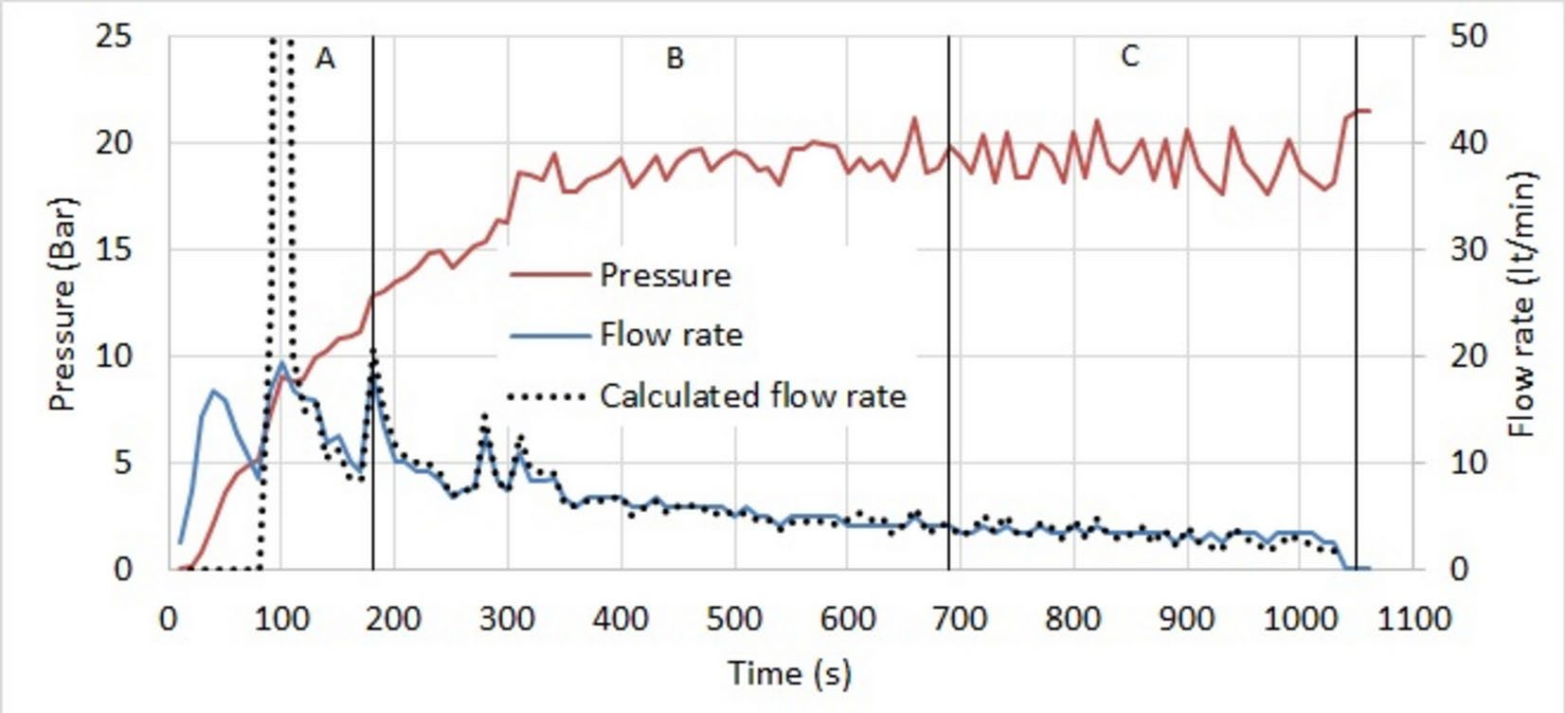
Grouting pressure, measured flow rate and calculated flow rate of stage 30–35.

The grouting pressure, measured flow rate and calculated flow rate of stage 30–35 are shown in Fig. 5. Three periods A, B and C are indicated in the figure. They differ by the number of conductive fractures and their average opening. They are indicated in Table 5 with the parameters of the back analysis. The number of conductive fractures in stage 30–35 is 5 at the beginning, then decreases to 2 and ends with 1. The average opening is 116 μm in the first two periods, and 131 μm in the third period. The hydraulic opening that was obtained from the water pressure test is 114 μm, in case there are 5 conductive fractures of equal opening, as in Table 3. The mean deviation is 6.3 μm. The viscosity is 1.4 mPas and is equal to the viscosity obtained in Stage 25–30 between 110 and 970 s. The yield stress is 7.2 Pa and is much larger than at stage 25–30. This is probably due to the narrowness of the fractures. The fractures of stage 30–35 are approximately 2d95 that renders the flow limited.

**Table 5:** Parameters of the back analysis of stage 30–35.

Period	–	A	B	C
Time	s	110–180	180–690	690–1060
Number of conductive fractures	–	5	2	1
Mean Opening	μm	116	116	131
Viscosity	mPas	1.4	1.4	1.4
Yield stress	Pa	7.2	7.2	7.2

## Consumption

In the previous section it was proven that the rock was permeated. In the present section the consumption is investigated. The opening of the fractures may explain the greater consumption of stage 25–30 in comparison to stage 30–35. The comparison must be done with certain rules, considering parameters that are common to the stages. The common characteristics are the viscosity of the mix, the length and diameter of the grout hole and the target. The target is the distance the mix must penetrate in the fractures to ensure an overlap with the mix from the neighboring grout holes. Generally, the spread must exceed half the distance between consecutive grout holes to form a continuous barrier. It was mentioned in Sect. "Comparison frame" that the target D is related to the flow rate limit Q_L_. The latter is the flow rate calculated at the target, leading to

5 $${\text{Q}}_{\text{L}}=\frac{2\uppi }{3\upmu }\frac{{\text{H}}^{3}\text{P}}{\text{ln}\left(1+\frac{\text{D}}{\text{a}}\right)}{\left(1-\frac{\text{cD}}{\text{PH}}\right)}^{2}\left(2+\frac{\text{cD}}{\text{PH}}\right)$$

**Fig. 6:**
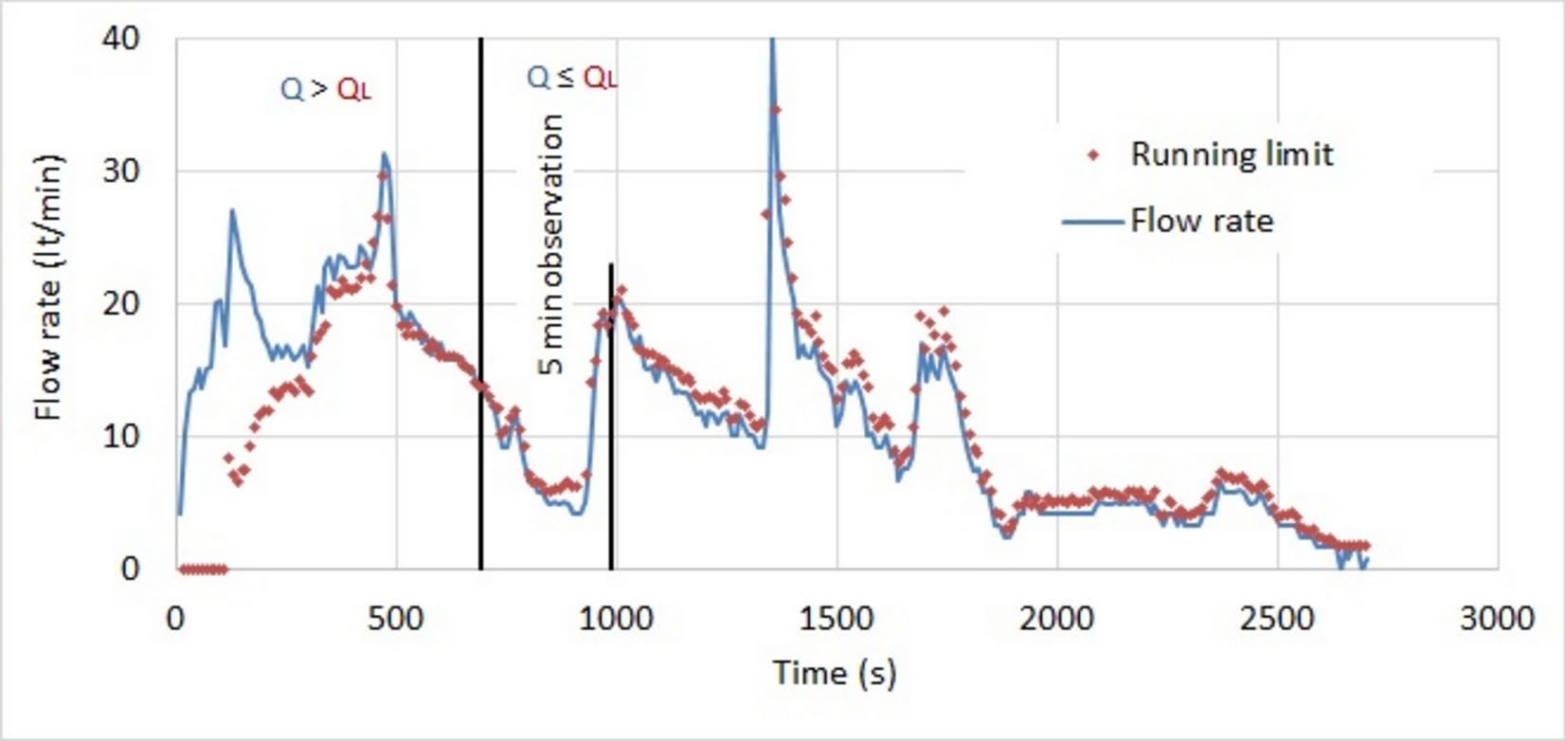
Measured flow rate at stage 25–30 and calculated running limit.

**Fig. 7:**
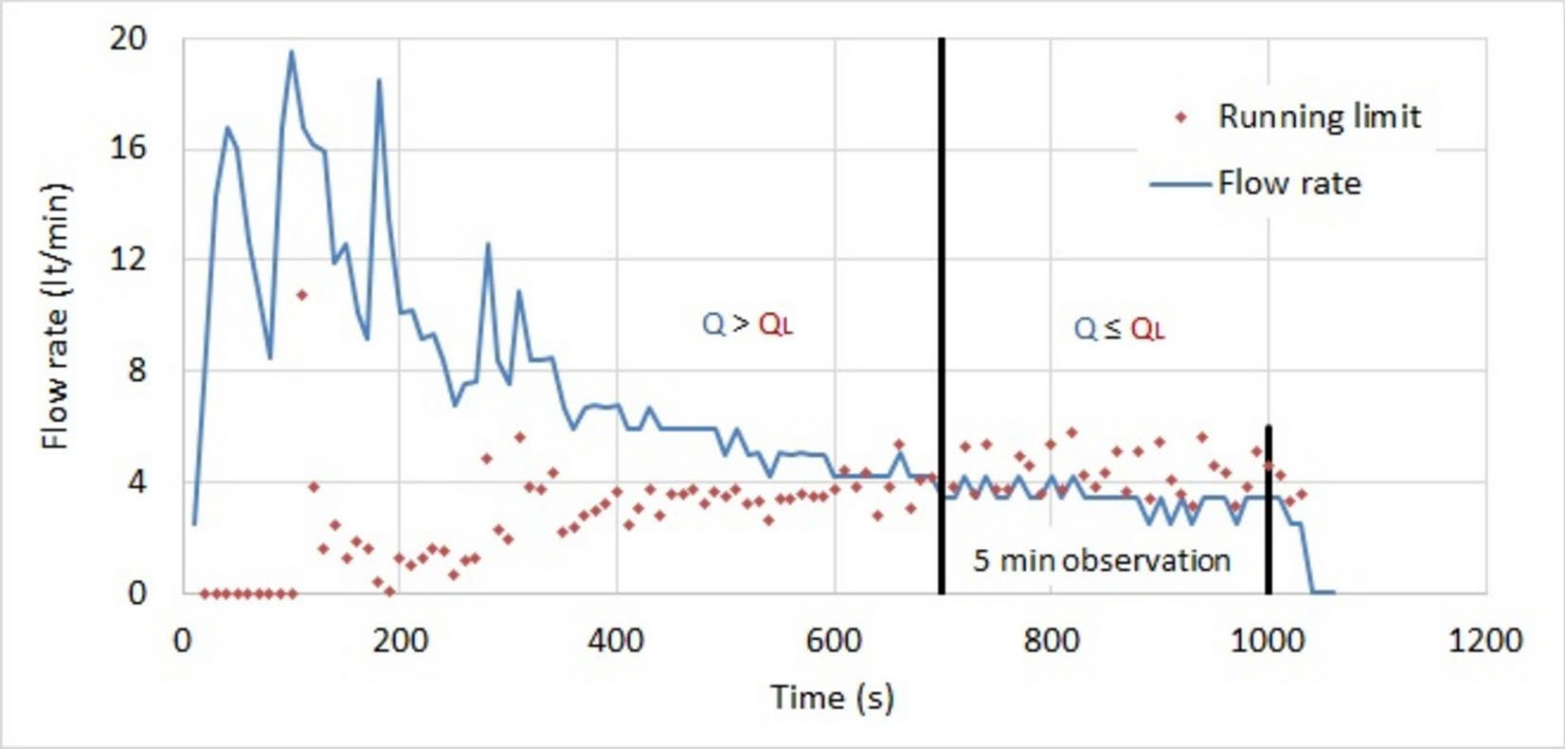
Measured flow rate at stage 30–35 and calculated running limit.

This relation remains valid when the pressure is slowly changing and is called the running limit^36^. The target is not a changing variable and is fixed by the designer. The running limit is calculated in real time, using the measured grouting pressure. In practice, the measured flow rate is greater than the running limit up to the moment the target is attained. At this moment, they are equal. After, the flow rate is smaller than the running limit. This is illustrated in Fig. 6 at stage 25–30 and Fig. 7 at stage 30–35. The graphics contain vertical lines, indicating where the target has been attained. When the target is reached then Q_L_ ≥ Q. An observation period of a few minutes is necessary for confirmation.

The target of the analyzed stages is set at 10m, which is more than half of the distance between the grout holes that are 12m distant. The overlapping of the disks has the shape of a convex lens with a thickness of 8m and a length between the cusps of 16m. The calculated running limit is compared to the measured flow rate in Fig. 6 for stage 25–30. The target is attained after 690s. Then a 5 min observation period is set for confirmation. The consumption is 216 lt at the target, 263 lt at the end of the observation period and 519 lt at the end of the grouting. The excess consumption of grout is 265lt, which is the difference between the consumptions at the end of the grouting and the end of the observation period.

For stage 30–35, the target is attained after 710s. The consumption is 98 lt at the target, 116 lt at the end of the observation period and 117 lt at the end of the grouting. The excess consumption of grout is one liter, which is less than 1% of the grouted quantity.

Table 6 shows the grout consumption of both stages. At the target, the consumption of stage 25–30 is 2.2 times that of stage 30–35, and at the end of the observation period it increases to 2.6. This proves that permeated fractures may have contrary absorption and consumption. This confirmation does not completely explain the difference between their total consumptions. The total consumption of stage 25–30 is 4.4 times that of stage 30–35. This is due to an over consumption, caused by ignoring to stop at the end of the observation period. The excess consumption of grout in stage 25–30 is higher than the consumption at the moment the target has been attained.

**Table 6:** Consumption at the target, 5 min later and the end of the grouting.

Stage	Target		End of the observation period	End of the grouting
	Time	Consumption	Consumption	Consumption
–	s	lt	lt	lt
25–30	690	216	263	519
30–35	710	98	116	117

## Conclusion

Contrary trends of water absorption and mix consumption are not all due to fracturing or fine fractures that cannot be grouted. Contrary trends also appear in permeated fractures and are due to the distribution of the number of fractures and their openings. This has been proven by a back analysis of grouting data at Sardasht dam. The comparison between the consumption data was established using the same target, the same mix and stages of equal lengths and diameters. It was deduced that over consumption of grout can occur when ignoring the target and failing to stop at the end of the observation period.

The analysis of the water pressure tests and grouting is useful for many purposes. On the one hand, it allows to update the investigations on the rock mass and its fractures, and particularly their conductivity, absorption and consumption. This information contributes to improve the characterization of the conductive zones that were identified during the preliminary study. On the other hand, it allows to adjust the grouting procedure in order to optimize the grouting works, according to the observational method.

Grout consumption analysis requires information regarding the number of fractures and their characteristics. The data were derived from water pressure tests and inspection in the borehole. The analysis needs a framework for the comparison with a target and an indicator that signals when the target is attained. This was done considering the minimal flow criterion and the running limit. The consumption analysis can be adapted and integrated into any rock grouting project, and coded to be run in real time. This is a means to optimize the grouting work and avoid overconsumption in compliance with sustainability.

## Data Availability

The data sets are available from M.H.N. on reasonable request.

## List of symbols

a: Radius of the grout hole
c: Yield stress of cement mix
D: Target; distance from the grout hole the mix must penetrate
d: Spread; penetration distance from the grout hole
d_95_: Size of the sieve grid through which 95% of the cement’s particles pass:
H: Half opening of a fracture; the opening is 2H
k: Hydraulic conductivity
lt: Liter; one thousandth of a cubic meter
L: Stage length
Lu: Lugeon unit
n: Number of conductive fractures
N: Number of fractures
P: Grouting pressure
Q: Flow rate
Q_L_: Flow rate limit
t: Time
t_i_: Time at the i^th^ step of a discretization
V: Volume of the injected cement mix
V_i_: Volume at time t_i_
μ: Dynamic viscosity of cement mix
μ_w_: Dynamic viscosity of water
γ_w_: Specific weight of water
